# The Impact of Authentic Leadership on Organizational Citizenship Behaviors: The Mediating Role of Affective‐ and Cognitive-Based Trust

**DOI:** 10.3389/fpsyg.2020.01975

**Published:** 2020-09-10

**Authors:** Tahir Farid, Sadaf Iqbal, Asif Khan, Jianhong Ma, Amira Khattak, Muhammad Naseer Ud Din

**Affiliations:** ^1^Department of Applied Psychology and Behavioral Sciences, Zhejiang University, Hangzhou, China; ^2^Department of Tourism and Hotel Management, School of Management, Zhejiang University, Hangzhou, China; ^3^Department of Tourism and Hospitality, Hazara University, Mansehra, Pakistan; ^4^Department of Marketing, College of Business Administration, Prince Sultan University, Riyadh, Saudi Arabia; ^5^Department of Education and Psychology, Kohat University of Science and Technology, Kohat, Pakistan

**Keywords:** authentic leadership, affective-based trust, cognitive-based trust, organizational citizenship behavior, Pakistan

## Abstract

Authentic leadership has appeared as a significant field of research. Building on social exchange theory that explicates how individuals mutually mechanize reciprocation and eventually establish a trust-based relationship, we postulated a positive relationship between authentic leadership and followers’ organizational citizenship behaviors (OCBs). Based on a two-wave time lagged design, the data were obtained from 270 employees working in a private banking sector of Pakistan. We found that authentic leadership is positively associated with subordinate’s OCBs, as well as leads to a higher-level of both affective‐ and cognitive-based followers’ trust. The results also indicated that both affective‐ and cognitive-based trust positively mediated the relationship between authentic leadership and OCBs. The implications for theory and practice are discussed.

## Introduction

Over the last decade, numerous empirical studies have investigated the significant role of authentic leadership in contributing to the effective functioning of the organizations and influencing followers’ job outcomes (Walumbwa et al., 2008a, 2011; Alilyyani et al., 2018). The predominant models of authentic leadership reveal that the authentic leader shows his true self to his subordinates, which helps in building trust, cooperation, and fostering teamwork among them (Gardner et al., 2005). Besides, considering trust is an important factor influencing the relationship between the leadership and followers’ job outcomes, past scholars have examined a strong association of authentic leadership with employees’ job outcomes through trust (Clapp-Smith et al., 2009; Wong et al., 2010; Hassan and Ahmed, 2011; Wang and Hsieh, 2013; Agote et al., 2016). Dirks and Ferrin (2002, p. 623) in their meta-analytic review of trust in leaders urged researchers to take into account multiple dimensions of trust, including affect-based and cognition-based trust and “attempt to distinguish between the processes involved,” but only a handful number of studies have actually responded to this call (Schaubroeck et al., 2011; Newman et al., 2014; Zhu and Akhtar, 2014). In addition, as both types of trust are of different nature (Dirks and Ferrin, 2002), it is believed that they may have different influences on the relationship between leadership and followers’ job outcomes. We extended this limited stream of research by examining the mediating role of affective-based and cognitive-based trust on the relationship between authentic leadership and followers’ organizational citizenship behaviors (OCBs).

McAllister (1995) introduced two types of trust (affect-based trust and cognition-based trust). Affective-based trust is relational or exchange based on nature, and cognitive-based trust is related to leader characteristics, such as ability, reliability, and integrity (McAllister, 1995; Dirks and Ferrin, 2002; Schaubroeck et al., 2011). Both types of trust represent distinct functions. Affective-based trust is related to a social exchange process (Blau, 1964), indicating a sense of responsibility to reciprocate and emphasize emotional ties between two parties. Past studies indicate that affective-based trust is considered as a feeling of security and strength of the relationship (Johnson and Grayson, 2005). In contrast, cognitive-based trust is related to followers’ instrumental evaluation of their leaders’ character, action, and decisions (Mayer et al., 1995), which reduce their ambiguity and embedded risk in a categorized relationship (Dirks and Ferrin, 2002; Colquitt et al., 2012).

Past empirical research reveals a positive mediating effect of affective‐ and cognitive-based trust on the association between transformational leadership and team performance at the group level (Schaubroeck et al., 2011). Considering trust as a fundamental pillar of authentic leadership development (Avolio and Gardner, 2005), an attempt has been made to examine the mediating role of affective-based trust and cognitive-based trust by focusing on the association between authentic leadership and OCBs at individual levels. Originally, affective-based trust and cognitive-based trust were conceptualized as an individual level construct (McAllister, 1995). Based on previous literature, we also conceive that it is suitable to investigate the mediating role of affective‐ and cognitive-based trust at the individual level. In addition, a plethora of leadership studies have indicated differential effects at both group and individual levels (Dansereau et al., 1999; Waldman and Yammarino, 1999). Similarly, understanding the vital role of authentic leadership at the workplace, several investigations have been conducted on analyzing the positive association between authentic leadership and OCBs (Valsania et al., 2012; Joo and Jo, 2017; Iqbal et al., 2018). This study also aimed to examine the authentic leadership behavior and its effect on employees’ OCBs in the context of the banking sector of Pakistan.

The current study intended to extend the contemporary literature in the field of authentic leadership, OCBs, and trust in the following aspects. First, past research reveals calls for more empirical research to examine authentic leadership globally across different industries and disciplines (Gardner et al., 2011; Alilyyani et al., 2018). This study will fill the literature gap by examining the effect of authentic leadership on OCBs in the context of the banking sector of Pakistan. Second, mostly, the studies related to authentic leadership and OCBs are conducted in the Western cultural context (Gardner et al., 2005; Walumbwa et al., 2008b; Valsania et al., 2012). However, this study focuses on Pakistan to see the influence of authentic leadership on OCBs among the private banking sector employees. Third, previous studies have examined affective‐ and cognitive-based trust in the relationship between transformational leadership or ethical leadership and employees’ job outcomes (Newman et al., 2014; Zhu and Akhtar, 2014). However, to date, none of the studies have been conducted on examining the mediating role of affective‐ and cognitive-based trust in the association between authentic leadership and OCBs. This is a pioneer study that examined the mediating role of affective‐ and cognitive-based trust in the relationship between authentic leadership and OCBs. Besides, this is a two-way study in that authentic leadership will influence employee’s individual levels OCBs through two mediators, namely, affective‐ and cognitive-based trust. By investigating the process through which both dimensions of trust transmit the effects of authentic leadership on employees OCBs, this study extends the authentic leadership and trust literature in a new important direction. Finally, as banking sector is the fastest-growing business in Pakistan, the model presented in the study will be helpful for the human resource managers and top management in designing effective leadership development programs.

## Theory and Hypothesis

### Authentic Leadership and OCBs

Authentic leadership appeared as an important area of research in the field of organizational behavior. Avolio et al. (2004) noted that authentic leaders are those “who are deeply aware of how they think and behave and are perceived by others as being aware of their own and others values/moral perspectives, knowledge, and strengths; aware of the context in which they operate; and who are confident, hopeful, optimistic, resilient, and of high moral character” (p. 4).

Authentic leadership is generally categorized into four dimensions: self-awareness, balanced processing, relational transparency, and internalized moral perspectives (Walumbwa et al., 2008a). Self-awareness is related to the leaders’ behaviors that indicate their awareness of own strength and weaknesses (Kernis, 2003) and their values and beliefs (Avolio and Gardner, 2005). Balanced processing refers to the leader’s ability to be unbiased in considering all relevant information before reaching any decisions (Leroy et al., 2012). In relational transparency, the leaders present their true selves to their followers, which help in cooperation, building trust, and fostering teamwork among coworkers (Gardner et al., 2005). An internalized moral perspective is related to leaders’ moral values and beliefs that are compatible with their behaviors (Walumbwa et al., 2008a).

Moreover, Organ et al. (2005, p. 3), defined OCBs as “individual behavior that is discretionary, not directly or explicitly recognized by the formal reward system, and in the aggregate promotes the efficient and effective functioning of the organization.” Tyler (2001) discussed OCB-related behaviors of the followers as the willingness to engage in extra-role behaviors that is beneficial for the organization (proactive behavior). The organizational management desires to enhance the OCB-related behaviors among employees to create dynamic workplace culture, as well as to improve the organizational productivity and maintain its sustainability (Lin and Ho, 2010). Past studies on OCBs identify it as a positive and productive behavior encouraged by supervisors and important for employees of the organizations (Vigoda-Gadot and Beeri, 2011). The pertinent literature also reveals that authentic leadership forms a positive, transparent, and fair atmosphere that affects employees’ willingness to engage in citizenship behavior (Walumbwa et al., 2008b). Joo and Jo (2017) found a positive association between authentic leadership and OCBs among employees of private sector organizations in Korea. Besides, Walumbwa et al. (2010) reported a significant positive influence of authentic leadership on followers’ OCBs in a telecom sector of China. In addition, Iqbal et al. (2018) found a positive association between authentic leadership and employees’ OCBs among the employees of the banking sector in Pakistan. Leadership translates proactive behaviors by providing specific directives and encouraging followers to cooperate by engaging in extra-role behaviors (Tyler, 2001; Wang and Hsieh, 2013).

The association between authentic leadership and OCBs can also be described from the lens of social exchange theory (Blau, 1964). Exchange theory suggests that “mutual reciprocation is the most basic form of human interaction” (Blau, 1964). This theory proposes that when followers perceive their leaders as authentic, they develop a strong sense of obligation and reciprocate by engaging more in citizenship behaviors. Social exchange theory also suggests that individuals’ orientation toward organization reflects their opinions about the favorability of the exchange of resources and efforts between them and the organization (Tyler, 2001). Thus, the social exchange theory paradigms and the discussion of authentic leadership and OCBs lead us to posit the following hypothesis:

*Hypothesis 1*: Authentic leadership is positively associated with follower’s OCBs.

### Authentic Leadership and Trust

Dirks and Ferrin (2002), in their meta-analytic review, conceptualized two different types of trust in leadership, such as affective‐ and cognitive-based trust. Affective-based trust stresses on the nature of leader and subordinate relationship (Konovsky and Pugh, 1994; Yang and Mossholder, 2010). Affective-based trust is operationalized as a social and emotional exchange-based relationship among leaders, and followers operate on the basis of care, concern, and mutual obligation (Dirks and Ferrin, 2002). The subordinates who experience respect, care, and concern from their leader reciprocate by showing more desired behaviors and emotional attachment. Hence, leaders’ positive behaviors representing care and concern may reveal that leaders will reciprocate with a balanced exchange and will take care of their subordinates’ needs (Dirks and Skarlicki, 2009; Colquitt et al., 2012).

Besides, cognitive-based trust approach is concerned with the leader character. Dirks and Ferrin (2002, p. 612) observed that “trust-related concerns about a leader’s character are important because the leader may have authority to make decisions that have a significant impact on a follower and the follower’s ability to achieve his/her goals (e.g., promotions, pay, work assignments, layoffs).” Subordinates may also cooperate with their leaders to maximize the personal benefits, but “choosing to do so also opens the door to potential exploitation” (Colquitt et al., 2012, p. 5). These types of perceptions and circumstances create a sense of ambiguity and risk among the subordinates, whether to cooperate or not with leaders. Subordinates reduce such ambiguity and risk by enhancing their predictability of leaders’ characteristics and by rationally considering their past experiences with the leaders (Molm et al., 2007).

Employees’ inclination to trust a leader is influenced by the actions and character of the leader (Heyns and Rothmann, 2015). Authentic leaders are the leaders who indicate authenticity and have the ability to enhance respect and dignity, integrity, and trust among followers (Bamford et al., 2013). Gardner et al. (2005) also argue that authentic leader has the ability to be aware of his strengths and weaknesses and to present one’s true and core self to other people and assist in building trust and cooperation and nurturing teamwork among colleagues. Such behavior of the authentic leader fosters the development of affective-based trust.

**Figure 1:**
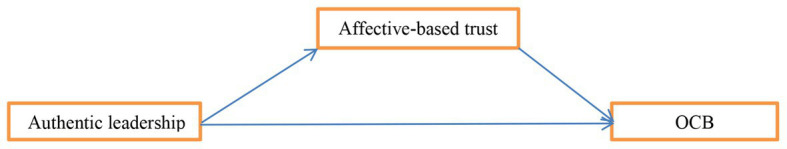
The relationship between authentic leadership and organizational citizenship behaviors (OCBs), authentic leadership, and affective-based trust, affective-based trust, and OCBs, as well as the mediating effect of affective-based trust on the relationship between authentic leadership and OCBs.

**Figure 2:**
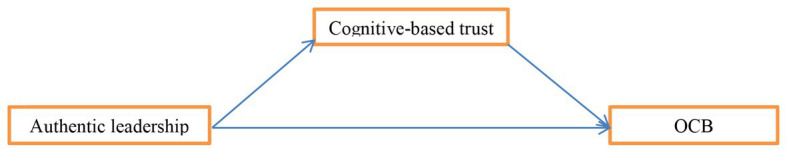
The relationship between authentic leadership and cognitive-based trust, cognitive-based trust, and OCBs, as well as the mediating effect of cognitive-based trust on the relationship between authentic leadership and OCBs.

Moreover, the relationship between authentic leadership and both affective‐ and cognitive-based trust can also be explained in the light of social exchange theory (Blau, 1964), drawing on the social exchange perspective when the followers’ beliefs about leader authenticity, competency, and honesty signal that the leader is the appropriate partner with whom to engage in a social exchange relationship, which is the characteristic of cognitive-based trust (McAllister, 1995). Moreover, empirical evidence reveals a positive relationship between transformational leadership and followers’ cognitive-based trust (Den Hartog, 2003; Newman et al., 2014; Zhu and Akhtar, 2014). Previous studies have also examined McAllister’s model of affective‐ and cognitive-based trust on the relationship between transformational leadership or ethical leadership and employees’ job outcomes (McAllister, 1995; Newman et al., 2014; Zhu and Akhtar, 2014). This study also followed model of trust of McAllister (1995) and examined the direct effect of authentic leadership on affective‐ and cognitive-based trust. Hence, based on the mentioned discussion on the relationships between authentic leadership, affective, and cognitive-based trust (Figures 1, 2), we assume the following hypotheses:

*Hypothesis 2*: Authentic leadership is positively associated with affective-based trust.
*Hypothesis 3*: Authentic leadership is positively associated with cognitive-based trust.

### Trust and Organizational Citizenship Behavior

Recent literature suggests a positive association between affective-based trust, cognitive-based trust, and citizenship behaviors (Zhu et al., 2013; Newman et al., 2014). A study conducted by Zhu et al. (2013) reveals that the relationship between affective-based trust and OCBs is stronger than the association between cognitive-based trust and OCBs. In addition, Newman et al. (2014) collected data from employees in a company in Southeast China; their findings reveal a positive association between affective-based trust and OCBs. As discussed in above literature, affective-based trust is more relational and exchange based that occurs when leaders are involved in an exchange relationship with their subordinates (Yang and Mossholder, 2010), and cognitive-based trust is related to leader competence and reliability (McAllister, 1995). We argue that along with effective-based trust, cognitive-based trust is also a crucial determinant for employees to enhance relationships with coworkers and leaders, which ultimately helps in enhancement of OCB-related behaviors among them. Thus, we conceive that both effective-based trust and cognitive-based trust positively influence employees OCB-related behaviors (Figures 1, 2). Based on the above discussion, we hypothesized the following:

*Hypothesis 4*: Effective-based trust is positively associated with followers’ OCBs.
*Hypothesis 5*: Cognitive-based trust is positively associated with followers’ OCBs.

### The Mediating Role Effective-Based Trust and Cognitive-Based Trust

In order to better understand how trust mediates the relationship between authentic leadership and followers’ OCBs, we chose a two-dimensional model of trust proposed by McAllister (1995), which differentiates between affective‐ and cognitive-based trust. We adopted the model of McAllister (1995) for two basic reasons. First, comparative to other existing models of trust, the two-dimensional model of trust of McAllister (1995) is more commonly used in leadership studies, specifically those that examine leadership styles to influence followers’ OCBs (Zhu et al., 2013; Newman et al., 2014). Second, the model of McAllister (1995) has appeared as a significant area for researchers and has been validated in various perspectives (Dirks and Ferrin, 2002; Wang et al., 2010; Yang and Mossholder, 2010).

McAllister (1995) model of trust basically distinguishes between two-dimensional affective‐ and cognitive-based trust. Affective-based trust, also called “trust from the heart” (Chua et al., 2008), is a relational-based approach developed on the basis of social and emotional exchanges, such as care and concern of one another and understanding of reciprocal sentiments (McAllister, 1995; Dirks and Ferrin, 2002; Zhu et al., 2013). Chua et al. (2008) labeled cognitive-based trust as “trust from the head” related to the assessment of one person’s fundamental characteristics, such as competence, reliability, and integrity by another person (Dirks and Ferrin, 2002).

In addition, as mentioned earlier, Avolio et al. (2004) believe that authentic leaders are those “who are deeply aware of how they think and behave and are perceived by others as being aware of their own and others’ values/moral perspectives, knowledge and strengths; aware of the context in which they operate; and who are confident, hopeful, optimistic, resilient, and of high moral character” (p. 4). Empirical evidence reveals the positive mediating role of trust in the association between authentic leadership and OCBs (Coxen et al., 2016). However, they have used a unidimensional construct of trust. Focusing on the distinctiveness of effective‐ and cognitive-based trust, we propose that both dimensions of trust positively mediate the relationship between authentic leadership and OCBs for distinct theoretical reasons. Authentic leadership may enhance followers’ affective-based trust by stimulating a sense of obligation, which helps in enhancing followers’ citizenship behavior. In addition, affective-based trust as a mediating variable also reveals the social exchange relationship accompanied by mutual obligations and relational bonds. Through the social exchange relationship, authentic leaders express care and concern to their followers, which help in strengthening the affective-based trust in the leader. Colquitt et al. (2012) found that affective-based trust increases the subordinate’s beliefs that the leader will respond with a balanced social exchange in relationships. Moreover, affective-based trust also strengthens the leader–follower social exchange relationship (Blau, 1964) and motivates subordinates to exceed their formal duties (Konovsky and Pugh, 1994; Colquitt et al., 2012).

Likewise, Dirks and Ferrin (2002) in their meta-analytic review suggest that cognitive-based trust in the leader may decrease the followers’ perceived risk of being vulnerable in a hierarchical relationship. In the presence of cognitive-based trust, the followers are not worried about leader exploitative behaviors (Mayer and Gavin, 2005). Furthermore, Dirks and Ferrin (2002, p. 623) in their meta-analytic review of trust in leaders also urged researchers to take into account multiple dimensions of trust, including affect-based and cognition-based trust and “attempt to distinguish between the processes involved,” but only a handful number of studies have responded to this call (Schaubroeck et al., 2011; Newman et al., 2014; Zhu and Akhtar, 2014). Hence, we also extended this limited stream of research by examining the mediating role of affective‐ and cognitive-based trust on the relationship between authentic leadership and followers’ OCBs (Figures 1, 2). Therefore, we hypothesized the following:

*Hypothesis 6*: Affective-based trust will have a positive mediating effect on the relationship between authentic leadership behaviors and followers’ OCBs.
*Hypothesis 7*: Cognitive-based trust will have a positive mediating effect on the relationship between authentic leadership behaviors and followers’ OCBs.

## Materials and Methods

Survey questionnaires were distributed among 270 employees working in different private banking sector organizations in Peshawar, Kohat, and Karak cities of Pakistan. The researchers visited different banks and discussed the study’s importance with every bank manager and encouraged staff members to participate in the study. After the formal approval of managers, the self-administered questionnaire was distributed among all the potential employees of the banks in two waves separately within a 1-month period. In the first wave, the followers provided their demographic information and rated the authentic leadership of their managers. In the second wave, the followers rated their OCBs, as well as affective‐ and cognitive-based trust in their managers. The researcher also ensured the confidentiality of the respondent’s responses.

By using convenient sampling technique, we distributed 350 questionnaires, but received 270 completed questionnaires. Of the total 270 respondents, majority of the respondents, 198 (73%), were male, and 72 (27%) were female. Most of the respondents, 153 (56%), were of the age between 21 and 30 years, 86 (32%) respondents were of the age between 31 and 40 years, and the remaining 31 (12%) were of the age between 41 and 50 years. In addition, majority of the respondents, 148 (55%) were married, 122 (45%) were unmarried, 85 (31%) held bachelor’s degree, 151 (56%) held a master’s degree, and the remaining 34 (13%) held MPhil or above level of education. Further, the majority of respondents, 170 (63%), had 1–5 years’ experience, 72 (27%) had 6–10 years’ experience, 21 (8%) had 11–15 years’ experience, and the remaining 7 (2%) had 16–20 years’ experience.

### Measures

All the scales were measured using five-point Likert scales ranging from strongly disagree to strongly agree.

### Authentic Leadership

Authentic leadership scale developed by Walumbwa et al. (2008a) was adopted for this study. The scale was composed of 16 items, and sample items included “My manager seeks feedback to improve interactions with others,” “My manager admits mistakes when they are made,” and “My manager demonstrates beliefs that are consistent with actions.” The reliability of the scale was 0.879.

### Organizational Citizenship Behavior

OCB was measured with eight items developed by Lee and Allen (2002). Examples included “I willingly give my time to help other staff members who have work-related problems” and “I show genuine concern and courtesy toward a staff member, even under the most difficult business or personal situations.” The Cronbach’s α of the scale was 0.871.

### Affective‐ and Cognitive-Based Trust

Affective‐ and cognitive-based trust were measured by using a self-reported scale developed by McAllister (1995). The scale of affective-based trust comprises five items, and cognitive-based trust consists of six items. The sample items of affective-based trust included “My manager and I have a sharing relationship. We can both freely share our ideas, feelings, and hopes,” “If I shared my problems with my manager, I know he would respond constructively and caringly.” The α value of the affective-based trust scale was 0.882. The sample items of cognitive-based trust included “My manager approaches his/her job with professionalism and dedication” and “I can rely on my manager not to make my job more difficult by careless work.” The α value of the cognitive-based trust scale was 0.829.

## Results

### Descriptive Statistics

The main characteristics of the sample, including means, standard deviations, and variable correlations, are shown in Table 1. The correlation between authentic leadership and followers’ OCBs (*r* = 0.147, *p* < 0.05) was found to be positive and significant, as expected; likewise, the correlation between authentic leadership and affective-based trust (*r* = 0.166, *p* < 0.001) was also found significant. The correlation between authentic leadership and cognitive-based trust was found significant (*r* = 0.198, *p* < 0.001). Besides, the correlation between OCBs and affective-based trust was found significant as expected (*r* = 0.140, *p* < 0.05). OCB correlations with cognitive-based trust were also found to be positive and significant as expected (*r* = 0.231, *p* < 0.001). Finally, the correlation between affective-based trust and cognitive-based trust was also found significant in the expected direction (*r* = 0.349, *p* < 0.001). Furthermore, we have also tested VIF and tolerance, which fall in the limits of threshold values (Hair et al., 2006).

**Table 1:** Descriptive statistics, mean, standard deviation (SD), and correlation of the variables.

	Mean	*SD*	1	2	3	4
Authentic leadership	3.3968	0.70595	1			
OCBs	3.7736	0.75607	0.147^*^	1		
Affective-based trust	3.5859	1.06080	0.166^**^	0.140^*^	1	
Cognitive-based trust	3.5710	1.00155	0.198^**^	0.231^**^	0.349^**^	1

*N* = 270;

* *p* < 0.05;

** *p* < 0.01;

*** *p* < 0.001.

### Regression Analysis of Authentic Leadership, Affective-Based Trust, Cognitive-Based Trust, and OCBs

Multiple linear regression was performed to examine the main hypotheses of the study. The results presented in Table 2 indicate the influence of authentic leadership and control variables, such as gender and age on the affective-based trust, cognitive-based trust, and OCBs.

**Table 2:** Regression analysis of authentic leadership, affective-based trust, cognitive-based trust, and OCBs.

Variables	Affective-based trust	Cognitive-based trust	OCBs
Constant			
Gender	0.196	0.376^**^	−0.073
Age	−0.083	0.056	0.092
Authentic leadership	0.249^**^	0.281^**^	0.158^*^
Affective-based trust			0.099^*^
Cognitive-based trust			0.174^***^
*R*^2^	0.027	0.039	0.022
∆*R*^2^	0.024	0.036	0.018
*F*	7.562^**^	10.972^**^	5.945^*^

*N* = 270;

* *p* < 0.05;

** *p* < 0.01;

*** *p* < 0.0001.

The result presented in Table 2 indicates the positive association of authentic leadership with followers’ OCBs (*β* = 0.158, *p* < 0.05), supporting our Hypothesis 1. Hypothesis 2 predicted the positive association of authentic leadership with affective-based trust. The results reveal that authentic leadership has a positive association with affective-based trust (*β* = 0.249, *p* < 0.001); hence, Hypothesis 2 is also fully supported. Hypothesis 3 predicted the positive association of authentic leadership with cognitive-based trust. The results reveal that authentic leadership has a positive association with cognitive-based trust (*β* = 0.281, *p* < 0.001); hence, Hypothesis 3 is also fully supported. Hypothesis 4 predicted a positive association of affective-based trust with followers’ OCBs. The result indicates that affective-based trust has a positive association with followers’ OCBs (*β* = 0.099, *p* < 0.05); hence, Hypothesis 4 is also fully supported. Similarly, Hypothesis 5 predicted a positive association of cognitive-based trust with followers’ OCBs. The result indicates that cognitive-based trust has a positive and significant association with followers’ OCBs (*β* = 0.174, *p* < 0.0001), fully supporting our Hypothesis 5.

### Mediation Analysis

The process program for SPSS developed by Hayes (2013) was used to analyze the mediating hypotheses. For this, we select model 4 from Hayes templates to find the mediating effect of affective-based trust and cognitive-based trust on the relationship authentic leadership followers’ OCBs. In addition, 95% correct bias CI with 5,000 bootstrapping procedures sample estimates was selected.

In Hypothesis 6, it is hypothesized that affective-based trust has a positive mediating influence on the association between authentic leadership and followers’ OCBs. The result (Table 3) reveals that affective-based trust positively and partially mediated the association between authentic leadership and followers’ OCBs (*b* = 0.137, *p* < 0.03), supporting our Hypothesis 6.

**Table 3:** Coefficient and bootstrapping for the mediation.

Testing paths	Unstandardized coefficient		*T*	Sig	Bootstrapping	
					LLCI	ULCI
	Coefficient	Standard error				
IV → M (a)	0.249	0.091	2.750	0.006	0.071	0.427
M → DV (b)	0.084	0.043	1.943	0.053	−0.001	0.170
IV → M → DV(c')	0.137	0.065	2.095	0.037	0.008	0.265
IV → DV (c)	0.158	0.065	2.438	0.015	0.030	0.285
Indirect effects	0.021	0.014			0.001	0.059

IV, authentic leadership; MV, affective-based trust; DV, OCBs; LLCI, lower level confidence interval; and ULCI, upper level confidence interval.

Similarly, in Hypothesis 7, it is hypothesized that cognitive-based trust has a positive mediating influence on the association between authentic leadership and followers’ OCBs. The results in Table 4 reveal that cognitive-based trust partially mediated the association between authentic leadership and followers’ OCBs (*b* = 0.113, *p* < 0.08), supporting our Hypothesis 7.

**Table 4:** Coefficient and bootstrapping for the mediation.

Testing paths	Unstandardized coefficient		*T*	Sig	Bootstrapping	
					LLCI	ULCI
	Coefficient	Standard error				
IV → M (a)	0.281	0.085	3.312	0.001	0.114	0.449
M → DV (b)	0.158	0.046	3.469	0.001	0.068	0.248
IV → M → DV(*c*')	0.113	0.065	1.750	0.081	−0.014	0.241
IV → DV (*c*)	0.158	0.065	2.438	0.015	0.030	0.285
Indirect effects	0.045	0.021			0.014	0.098

IV, authentic leadership; MV, cognitive-based trust; DV, OCBs; LLCI, lower level confidence interval; and ULCI, upper level confidence interval.

## Discussion

The present study explored the direct effect of authentic on followers’ OCBs as well as the indirect effect of authentic leadership on followers’ OCBs through affective-based trust and cognitive-based trust.

The present study reveals a positive association between authentic leadership and followers’ OCBs. As discussed in the previous section, authentic leadership has become the focus of attention among scholars because of its positive effects on employees as well as on organizational goal achievements (Walumbwa et al., 2008a, 2010, 2011), and they call for more empirical work (Gardner et al., 2011; Avolio and Walumbwa, 2014; Alilyyani et al., 2018). The current study filled this gap by examining the association between authentic leadership and followers’ OCBs in the context of the private banking sector of a developing country, Pakistan. In addition, in line with our expectations and previous research findings (Walumbwa et al., 2008a; Joo and Jo, 2017; Iqbal et al., 2018), the current study findings indicate a positive association between authentic leadership and followers’ OCBs supporting Hypothesis 1. Moreover, in line with our expectation, authentic leadership is found positively associated with affective-based trust and cognitive-based trust supporting our Hypotheses 2 and 3. Further, in agreement with previous research (Zhu and Akhtar, 2014), our findings also reveal that affective-based trust and cognitive-based trust are positively associated with followers’ OCBs, supporting our Hypotheses 4 and 5.

Besides, our results also support the social exchange theory (Blau, 1964), which stated that “mutual reciprocation is the most basic form of human interaction.” In the workplace, this theory proposes that when followers perceive their leaders as authentic, they develop a strong sense of obligation and reciprocate by engaging more in citizenship behaviors beyond their formal roles. Hence, our study findings also support the exchange base relationship between leaders and followers by showing the positive influence of authentic leadership behaviors on followers’ OCB-related behaviors.

This is a pioneer study that examined trust-based mechanism through which authentic leadership affects subordinates’ OCBs. In agreement with past researches in the area of ethical leadership (Newman et al., 2014) and transformational leadership (Zhu and Akhtar, 2014), our results also indicate that affective-based trust mediated the relationship between authentic leadership and subordinates’ OCBs supporting our Hypothesis 6. Besides, our study also makes an important contribution in the area of cognitive-based trust in explaining the relationship between authentic leadership and OCBs. Consistent with the previous researches in the area of leadership (Newman et al., 2014), we also found that cognitive-based trust mediated the relationship between authentic leadership and OCBs, supporting our Hypothesis 7. From a social exchange perspective, our study findings also indicate how both the dimensions of trust stimulate positive workplace relationship’s outcomes and play a vital role in developing the positive relationship between authentic leadership and followers’ OCBs. In other words, by taking into account the trust-based mechanism of McAllister (1995) to identify the relationship between authentic leadership and subordinates’ OCBs, our study makes a new contribution to the authentic leadership literature.

### Practical Implications, Limitations, and Future Research Suggestions

The results of the current study established the important role of authentic leadership in stimulating positive job outcomes through the development of affective‐ and cognitive-based trust.

The banking sector has appeared as the fastest-growing business sector because of the opening of new multinationals banks and advancement in technology. Therefore, the bank managers should confirm that the objectives of both management and employees are well-matched.

Based on findings, the top management should pay attention to the importance of authentic leadership style of managers and emphasis on leadership development programs. Considering the importance of authentic leadership, the top management should take into account the components of authentic leadership in developing strategies, evaluation, and selection procedures. For instance, they may give more importance to the training of both managers and their subordinates to advance their concern toward workplace issues. This results in the exhibition of more authentic behavior by leaders, as well as enhances the receptivity of subordinates to such behaviors.

The top management should establish development department and training at various levels. The professional in the field of human resource management who can assist the managers in employees training, career development, and motivation strategies should be hired. The findings of this study can be helpful in developing different strategies for employees to enhance the awareness among leaders and managers in the banking sector. Furthermore, the findings of the study focus on the need to develop followers’ feedback system about their managers to the top management.

In addition, in the leadership development program, the management should consider the importance of trust enhancement policies. The leaders and managers should share their personal experiences with the followers, which can play an important role in enhancing affective-based trust. Further, the leaders should demonstrate beliefs that are consistent with actions, as the action of the leaders at the workplace can develop followers’ perceptions of leader competence and reliability. Thus, the successful leadership program intended at nurturing affective‐ and cognitive-based trust can be increased by integrating these trust enhancing policies.

This study also has some limitations. First, the data are collected in two phases 1 month apart to test the hypotheses. Future studies should vary the time between surveys to see how far in time the influence of authentic leadership perceptions extends. Second, as the data were collected from a single source for instance banking sector, to avoid the possibility of common method biasness, we followed the recommendation of Podsakoff et al. (2003) for a questionnaire design. We have (a) kept respondents’ confidentiality and anonymity to reduce their assessment apprehension and make them less likely to edit their feedback to be more socially desirable, (b) confirmed psychological separation and methods of measurement by using by writing descriptive sentence to make it visible that the measurement of independent variable is not connected with or related to the measurement of the dependent variable. Third, the present study is conducted in the collectivistic culture of Pakistan; in the future, the study should be replicated in individualistic cultures in which interpersonal relationships are less important to organizational success. Fourth, as the data are collected from a single sector, for instance, banking sector, for its great generalizability, it can be extended to other organizations and cultures. Finally, the future researches are encouraged to consider other organizational variables such as employee well-being and creativity while focusing on authentic leadership‐ and trust-based mechanisms.

## Conclusion

This is a pioneer study that examines the trust-based mechanism underlying the relationship between authentic leadership and subordinates’ OCBs. It makes an important contribution to authentic leadership literature by providing the vital role of affective‐ and cognitive-based trust in influencing the relationship between authentic leadership and OCBs. This study confirms the vital role of social exchange theory (Blau, 1964) in integrating the relationship between authentic leadership, trust, and OCBs. Hopefully, the study findings will motivate scholars to examine in greater depth the trust-based mechanism by which authentic leaders influence subordinates’ workplace behaviors.

## Data Availability Statement

The raw data supporting the conclusions of this article will be made available by the authors, without undue reservation, to any qualified researcher.

## Ethics Statement

The studies involving human participants were reviewed and approved by the ethics commission of Zhejiang University. The patients/participants provided their written informed consent to participate in this study.

## Author Contributions

The first author (TF) and the second author (SI) equally contributed to writing the original draft, the conceptualization, data collection, formal analysis, and methodology. JM provided resources and administered the project. AKn, AKt, and MN reviewed and edited the paper. All authors contributed to the article and approved the submitted version.

### Conflict of Interest

The authors declare that the research was conducted in the absence of any commercial or financial relationships that could be construed as a potential conflict of interest.
