# Corrigendum: The Impact of Authentic Leadership on Organizational Citizenship Behaviors: The Mediating Role of Affective- and Cognitive-Based Trust

**DOI:** 10.3389/fpsyg.2020.605350

**Published:** 2020-10-22

**Authors:** Tahir Farid, Sadaf Iqbal, Asif Khan, Jianhong Ma, Amira Khattak, Muhammad Naseer Ud Din

**Affiliations:** ^1^Department of Applied Psychology and Behavioral Sciences, Zhejiang University, Hangzhou, China; ^2^Department of Tourism and Hotel Management, School of Management, Zhejiang University, Hangzhou, China; ^3^Department of Tourism and Hospitality, Hazara University, Mansehra, Pakistan; ^4^Department of Marketing, College of Business Administration, Prince Sultan University, Riyadh, Saudi Arabia; ^5^Department of Education and Psychology, Kohat University of Science and Technology, Kohat, Pakistan

**Keywords:** authentic leadership, affective-based trust, cognitive-based trust, organizational citizenship behavior, Pakistan

In the published article, there was an error in affiliation 4. Instead of “King Saud University”, it should be “Prince Sultan University.”

The authors apologize for this error and state that this does not change the scientific conclusions of the article in any way. The original article has been updated.

